# Experimental Study on Mechanical Properties and Pore Structure Deterioration of Concrete under Freeze–Thaw Cycles

**DOI:** 10.3390/ma14216568

**Published:** 2021-11-01

**Authors:** Kai Zhang, Jing Zhou, Zhigang Yin

**Affiliations:** 1 Institute of Earthquake Engineering, Faculty of Infrastructure Engineering, Dalian University of Technology, Dalian 116024, China; zhouj@dlut.edu.cn; 2 State Key Laboratory of Coastal and Offshore Engineering, Dalian University of Technology, Dalian 116024, China; 3 Laboratory of Applied Disaster Prevention in Water Conservation Engineering of Jilin Province, Changchun Institute of Technology, Changchun 130012, China

**Keywords:** concrete, mechanical properties, microscopic pore structure, freezing and thawing cycles, NMR technique

## Abstract

Understanding the evolution of mechanical properties and microscopic pore structure of concrete after freeze–thaw cycles is essential to assess the durability and safety of concrete structures. In this work, the degradation law of mechanical properties and damage characteristic of micro-structure of concrete with two water-cement ratios (w/c = 0.45 and 0.55) is investigated under the condition of freezing–thawing cycles. The influence of loading strain rate on dynamic compressive strength is studied. The microscopic pore structure after frost damage is measured by low-field nuclear magnetic resonance (LF-NMR) technique. Then, a damage model based on the porosity variation is established to quantitatively describe the degradation law of macroscopic mechanical properties. The test results show that the relative dynamic modulus of elasticity (RDME), dynamic compressive strength, flexural strength, and splitting tensile strength of concrete decrease with the increase of freeze–thaw cycles. Empirical relations of concrete dynamic increase factor (DIF) under the action of freeze–thaw cycles are proposed. Moreover, the experimental results of NMR indicate that the porosity as well as the proportion of meso-pores and macro-pores of concrete gradually increased with the increasing of freeze–thaw cycles. The research results can provide reference and experimental support for the anti-frost design theory and durability life prediction of hydraulic concrete structures in cold regions.

## 1. Introduction

Material degradation caused by freeze–thaw cycles is one of the major durability problems of concrete structures in cold regions [1,2,3], where many concrete structures located at the waterline fluctuation are suffer from severe deterioration, such as concrete dams, hydraulic power plants, and offshore structures [4,5]. Icold Committee on Deterioration of Dams and Reservoirs (1984) reported that 19% of reported cases of dam deterioration are attributed to freezing and thawing cycles [6]. Especially in the northeast of China, nearly all hydraulic concrete structures suffer from freeze–thaw damage. The influential factors of material degradation during freezing and thawing are w/c ratios, porosity, air-content, cement, and aggregate. Surface scaling and the internal damage are the two typical characteristics under the freezing–thawing condition, which are caused by progressive expansion of the cement paste matrix from periodic freezing and thawing cycles [7,8].

In the past few decades, numerous studies have been conducted on the evolution laws of mechanical deterioration [9,10] and damage mechanisms [11,12,13] of concrete caused by freeze–thaw cycles. Less attention has been given to the problem of assessing the dynamic mechanical properties after frost damage. Additionally, concrete is also a strain rate-sensitive material. Its strength and deformation characteristics under dynamic loading are different from those under static loading, and these differences are critical factors for the safety of the structure under certain conditions [14,15,16,17]. Xiao et al. [18] investigated the effect of strain rate and loading history on the dynamic compressive strength and damage evolution of concrete. Li et al. [19] investigated the impact dynamic properties of concrete after freeze–thaw using the SHPB technique and further explained the damage process and mechanism of freeze–thaw of concrete. Han et al. [20] analyzed the dynamic mechanical properties of concrete after frost damage. However, the experiment contained only one type of water–cement ratio concrete, and the equations proposed are only suitable for their own experimental data, which is not universal. Therefore, it is necessary to further study the evolution of dynamic mechanical properties of concrete with different water–cement ratios after freeze–thaw.

The porosity and pore size distribution (PSD) are significant parameters of the pore structure, which directly affect the permeability, mechanical properties, and durability of concrete [21,22,23]. Kashif et al. [24,25] concluded that the incorporation of 0.03% graphene oxide (GO) can significantly improve all mechanical properties and reduce microcracks or porosity of the cement composites. In this regard, several researchers have conducted a large number of experimental and theoretical studies on the expansion law of concrete internal pores after freeze–thaw cycles. Zhang et al. [26] and Yan et al. [27] discussed the evolution of pore structure and PSD of cement slurries and metakaolin-based geopolymers materials during freeze–thaw cycles using the MIP technique, respectively. Fan et al. [28] and Wang et al. [29] analyzed the microscopic characteristics of concrete with different mineral admixtures after freeze–thaw cycles based on SEM. Yuan et al. [30] conducted an experimental study on the changes of concrete pores after frost damage using X-ray CT techniques. However, each of the above methods of testing the internal pore structure of concrete has its own limitations. For MIP tests, the sample needs to be vacuum dried, and the mercury is invasive and can damage the original pore structure of the sample. Thus, it is difficult to obtain the characteristics of continuous changes in pore structure. The SEM method has a limited test range for pore size. In addition, using X-ray CT techniques to test the pore structure of cement materials usually takes a long time and is expensive.

Currently, LF-NMR technique is gradually and widely used as a new and rapid method to detect the microstructure of rocks [31], concrete [32,33,34], and other porous media [35]. The method is based on water as the medium and has many advantages such as non-destructive, non-invasive, fast testing speed, and accurate results, which has become a very powerful way to monitor the internal micro-structural changes. Wang et al. [36] analyzed the pore structure damage propagation characteristics of natural pumice light aggregate concrete after freeze–thaw using NMR technique. However, the relationship between the porosity obtained from NMR and the mechanical properties of concrete is less studied.

The goal of this study is to investigate the mechanical properties and pore structure degradation of concrete with different water–cement (w/c) ratios under the action of freezing–thawing cycles. The attenuation rules of mass loss rate, RDME, compressive strength, flexural strength, and splitting tensile strength after frost damage are analyzed. The dynamic compressive properties of concrete are experimentally investigated at the strain loading rates ranging from 10^−5^/s to 10^−2^/s. The porosity, transverse relaxation time *T*_2_ distribution, and PSD after freeze–thaw cycles are obtained. The empirical relations of concrete DIF under the action of freeze–thaw cycles are proposed. The relationship between NMR porosity damage and strength are established, which can provide data support and reference for the durability prediction and safety assessment of concrete structures in cold regions.

## 2. Materials and Methods

### 2.1. Material Properties 

**Figure 1:**
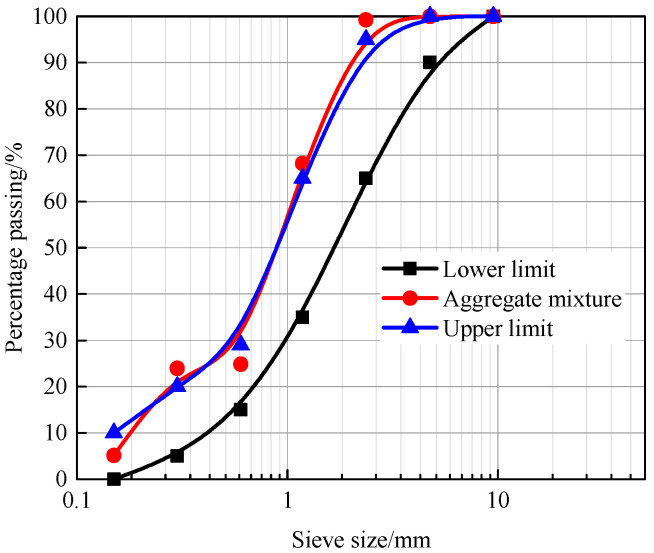
Particle size distributions of the natural river sand.

Ordinary Portland cement P.O 42.5 was used for preparing concrete specimens, which is produced by Yatai Group Co., Ltd (Jilin, China). The density of the cement is 3105 kg/m^3^, the specific surface area is 330 m^2^/kg. The initial and final setting times are 155 and 215 min, respectively. The compressive strengths at 7 and 28 days are 26.2 and 48.8 MPa, the flexural strengths at 7 and 28 days are 5.6 and 8.5 MPa, respectively. The common natural river sand was used with a fineness modulus of 2.66, and its particle size distributions are illustrated in Figure 1. Crushed stones with apparent density of 2710 kg/m^3^ and continuous gradation from 5 to 31.5 mm were chosen as coarse aggregates. In addition, a modified FDN superplasticizer was used with a mixing amount of 0.8% (by the weight of cement), the pH value of the FDN superplasticizer (Xingzhenghe chemical Co., Ltd, Shenyang, China) was 8.19, and the water reducing rate was 21%.

### 2.2. Mixture Proportion and Preparation of Specimens

Previous studies have shown that the water–cement ratio and loading rate have a great influence on the compressive strength of concrete. Therefore, two different water–cement ratio (w/c) concrete mixtures were designed and prepared in the laboratory conditions. The mix proportions of concrete are given in Table 1. The concrete mixtures were carried out in a horizontal shaft blender. The mixing process of concrete specimens is described below. Firstly, coarse aggregate and river sand were poured into the blender and mix thoroughly for about 60 s. Then, 40% of total mixing water was gradually added to the blender and stirred for 60 s, the cement was accurately weighed and added to the blender, and then they were combined and stirred for 60 s. After that, the remaining 60% of total water with the dissolved FDN superplasticiser were added to the mixture and stirred for 120 s. Finally, the concrete mixtures were poured into the plastic molds in three layers, and then placed on the shaking table and vibrated for 20–30 s. All the specimens were placed in the laboratory for 24 h. Then they were demolded and cured in standard curing conditions (the temperature was about 20 ± 2 °C and the relative humidity of 95% RH) until the days for testing.

**Figure 2:**
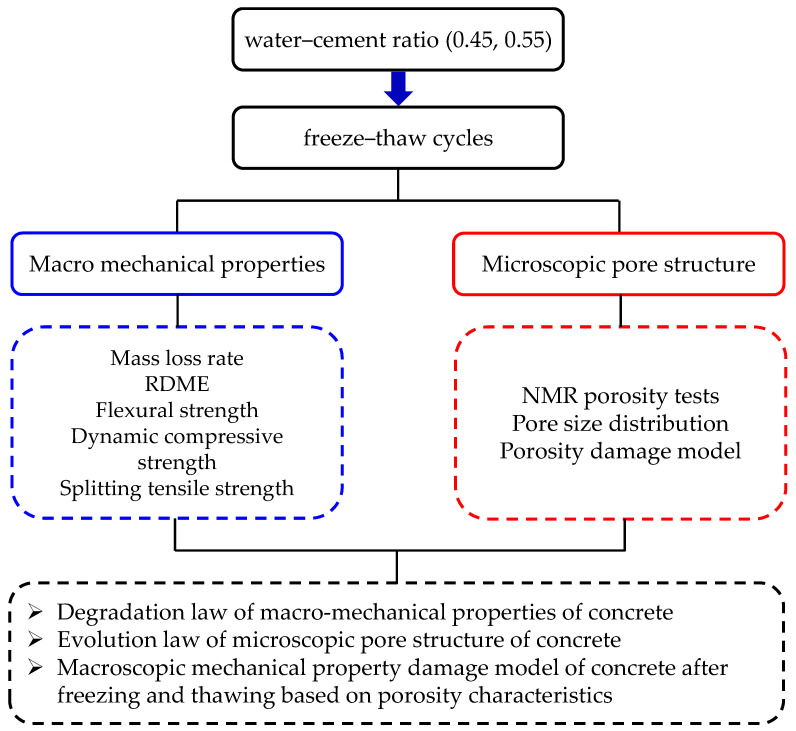
Flow chart of the tests.

The flow chart is shown in Figure 2. For each mixture, there were 12 prism specimens with dimensions of 100 × 100 × 400 mm for rapid freeze–thaw test, three of which were used to measure the mass loss, RDME after every 25 freeze–thaw cycles. In addition, the remaining prism specimens were used to measure the flexural strength, and then drill cylinder cores (diameter × height, 50 × 100 mm) to measure the porosity after different freeze–thaw cycles. Additionally, the cube specimens were fabricated to obtain compressive strength. For each mixture, the strength tests were conducted on three parallel specimens and the averaged results were presented.

### 2.3. Experiment Methods

#### 2.3.1. Freeze–Thaw Test

**Figure 3:**
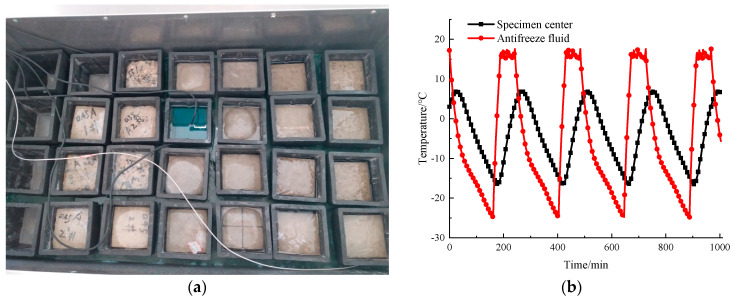
Rapid freeze–thaw testing machine and temperature change curve. (**a**) Rapid freeze–thaw testing machine, (**b**) Temperature change curve.

The rapid freeze–thaw method used in this test was based on the Chinese national standard GB/T 50082-2009 [37], which is similar to the standard ASTM C666/C666M-15 [38]. Each freeze–thaw cycle was completed within 3–4 h. During each cycle, the maximum temperature and minimum temperature at the center of the specimen was controlled at 5 ± 2 °C and −18 ± 2 °C, respectively. The rapid freeze–thaw testing machine and temperature change curve of the freeze and thaw cycles are plotted in Figure 3a,b, respectively.

The mass loss rate ($\Delta W_{N}$), RDME were measured to evaluate the deterioration of the specimens under freezing and thawing cycles. After every 25 freeze–thaw cycles, the specimens were removed from the freeze–thaw chamber, and then physical change, dynamic modulus of elasticity, compressive strength were conducted. In this test, the specimen was considered to be a failure if the RDME loss exceeded 40% or the mass loss rate exceeded 5%. Consequently, the $\Delta W_{N}$ of the specimen was calculated as follows:

(1) $$\Delta W_{N}=(\frac{W_{0}-W_{N}}{W_{0}})\times 100\%$$

where $\Delta W_{N}$ is the average mass loss rate of the three prismatic specimens after freeze–thaw cycles (%); $W_{N}$ is the mass of the specimen after *N* freeze–thaw cycles (kg); $W_{0}$ is the initial mass of the specimens (kg).

The RDME of the prism concrete specimens were measured by DT-18 testing instrument (Naijiuweiye, Beijing, China). The relative dynamic modulus ($\Delta E_{r}$) was calculated by Equation (2) to evaluate the internal damage of the concrete specimens, which is defined as,

(2) $$\Delta E_{r}=\frac{E_{N}}{E_{0}}\times 100\%=\frac{f_{N}^{2}}{f_{0}^{2}}\times 100\%$$

where $\Delta E_{r}$ represents the RDME of the concrete specimen after N freeze–thaw cycles, %; $E_{0}$ is the initial dynamic elastic modulus of concrete specimen, while $E_{N}$ is the dynamic elastic modulus of the specimen after N freeze–thaw times (GPa); $f_{0}$, $f_{N}$ is the transverse fundamental frequency of the specimen before and after freeze–thaw cycles (Hz), respectively.

#### 2.3.2. Compressive Strength

In order to study the evolution of dynamic mechanical properties of concrete subjected to freeze–thaw, uniaxial compressive strength tests were conducted on cube specimens under different loading rates. In this test, compressive strength tests of the cubic specimens after each 25 freeze–thaw cycles were conducted, according to Chinese standard specification GB/T 50081-2019 [39], which is similar to the Standard test method for compressive strength of cylindrical concrete specimens (ASTM C39/C39M−18) [40]. The loading was carried out in the form of displacement control. The loading rates were 0.06, 0.6, 6, and 60 mm/min, respectively. The strain rates corresponding to the four loading rates were 1 × 10^−5^/s, 1 × 10^−4^/s, 1 × 10^−3^/s, 1 × 10^−2^/s, respectively.

**Figure 4:**
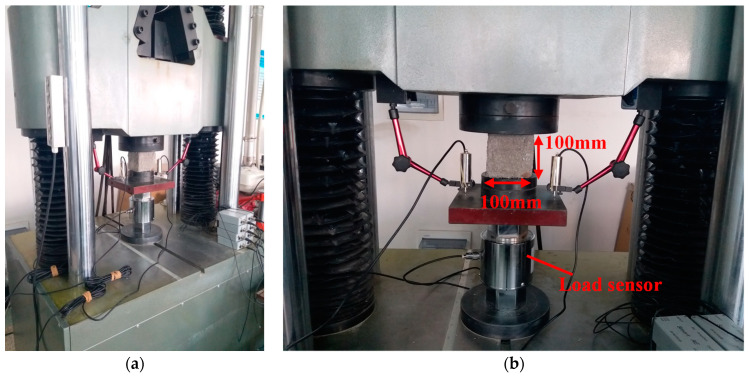
Schematic diagram for compression test of cubic specimen. (**a**) WAW–2000 kN test machine, (**b**) Test setup for cubic compressive strength.

The mechanical tests were conducted on a WAW-2000 kN microcomputer controlled electro-hydraulic servo test system (Keda, Changchun, China) Two displacement sensors were placed on the two sides of the cube specimen to record their deformation during the loading process. Figure 4 shows the diagram for uniaxial compression test. Before formal loading, the cubic specimen was first preloaded with 5 kN to ensure that the loading plate is in full contact with the surface of the specimen. After that, the loading was carried out according to the determined loading rate until the specimens failed.

#### 2.3.3. Flexural Strength and Splitting Tensile Strength

**Figure 5:**
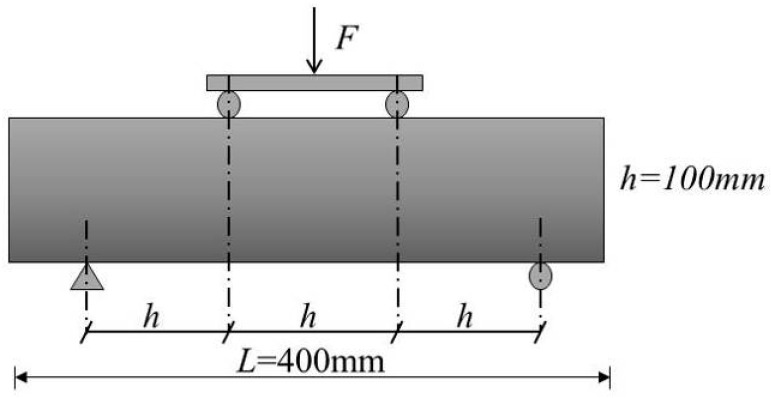
Schematic diagram of flexural strength test.

The flexural strength and splitting tensile strength of concrete are another key property for evaluating the durability of concrete. In this test, the flexural strength and splitting tensile strength of the concrete specimens with different w/c ratios under freeze–thaw cycles were tested, according to Chinese standard specification GB/T 50081-2019 [39] and ASTM C78/C78M-18 [41]. The flexural strength test loading schematic is shown in Figure 5.

To better evaluate the effect of freeze–thaw cycles on the frost durability of concrete with different water–cement ratios, the loss of flexural strength under freeze–thaw cycles (0–225 cycles) was calculated according to Equation (3) based on the test results.

(3) $$\Delta f_{f}=\frac{f_{f,0}-f_{f,N}}{f_{f,0}}\times 100\%$$

where $\Delta f_{f}$ is the flexural strength loss of specimens after *N* freezing and thawing cycles; $f_{f,0}$ is the initial flexural strength, MPa; $f_{f,N}$ is the flexural strength after *N* freeze–thaw cycles, MPa.

#### 2.3.4. Low Field Nuclear Magnetic Resonance Tests

**Figure 6:**
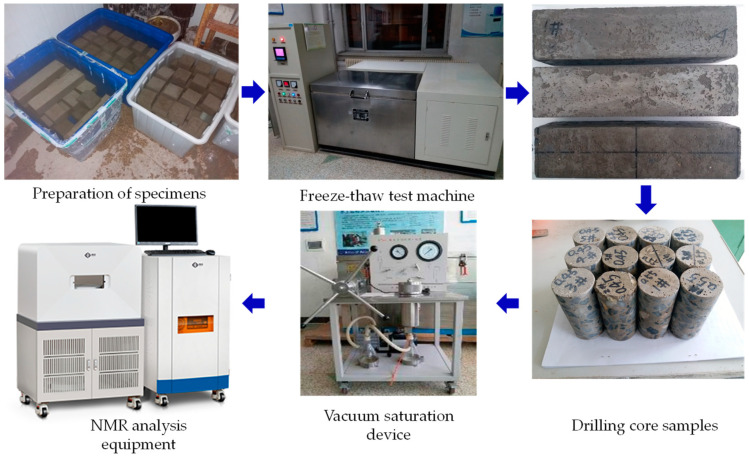
Flow chart of NMR test.

In this experiment, a MesoMR23-060H-I instrument (Niumag, Shanghai, China) was used to test the pore structure of concrete drill core samples after frost damage. The NMR analysis equipment has a resonance frequency of 21.3 MHz, a magnet strength of 0.5 ± 0.08 T, a coil diameter of 60 mm. Additionally, the measurement parameters were set as follows: P1 = 13.00 μs, P2 = 26.00 μs, SW = 200 kHz, TR = 1000 ms, NS = 32, Echo spacing time = 0.26 μs, Echo count = 18,000. During the NMR tests, the temperature of the magnet and probe assembly was maintained at 32 ± 0.01 °C. The room temperature was set at 25 ± 1 °C by air conditioning. The size of concrete sample used for NMR test was 50 × 100 mm (diameter × height). Prior to the test, the drill core sample was wiped to clean the surface mortar and was vacuum-saturated with water for 24 h by using a ZYB-II vacuum saturator (Huaxing, Nantong, China). Compared with CT technique, the LF-NMR test needs more preparation and time. The experimental procedure is shown in Figure 6.

According to the theory of NMR, the Carr–Purcell–Meiboom–Gill (CPMG) sequence is usually used to test the relaxation time information of cement-based materials [32,34,35]. Based on the NMR relaxation mechanism, for porous media, the transverse relaxation rate can be simply described as follows.

(4) $$\frac{1}{T_{2}}=\rho_{2}\left(\frac{S}{V}\right)=\rho_{2}\frac{F_{s}}{R}$$

where, *T*_2_ is the transverse relaxation time of water in the pore, ms; *ρ*_2_ is the strength of transverse surface relaxation (μm/ms); *S*/*V* is the surface–volume ratio of the pores; *F_s_* is the shape scale factor; *R* is the pore radius.

It can be seen from Equation (4) that the transverse relaxation time is only related to *S*/*V*, and *T*_2_ is positively correlated with pore radius. The short relaxation times have smaller pore sizes, while longer times have larger pore sizes. Therefore, the transverse relaxation time *T*_2_ distribution curve is consistent with the pore size distribution curve, which can reflect the distribution of moisture in the internal pores of concrete.

## 3. Experimental Results 

### 3.1. Appearance Change

#### 3.1.1. Typical Failure Characteristics of Prism Specimen

**Figure 7:**
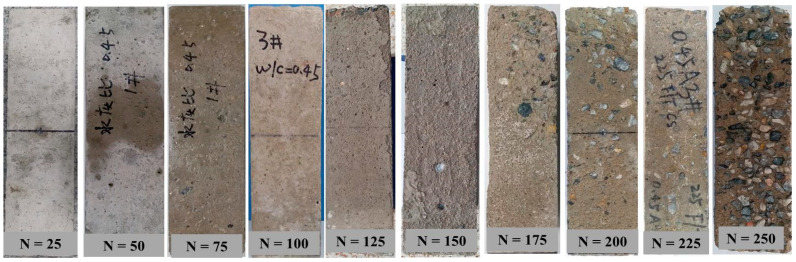
Typical failure characteristics of concrete specimens under different freeze–thaw cycles.

Figure 7 demonstrates the typical failure characteristics of concrete specimens under different freeze–thaw cycles. As can be seen in this figure, with the increasing number of freeze–thaw cycles, the surface of the concrete prismatic specimen becomes less flat, the mortar falls off, and the phenomenon of exposed aggregates becomes more and more serious. Before 75 cycles of freeze–thaw, there is no obvious change in the appearance of the prism specimens, the shape and size of the specimens maintained its integrity. After nearly 125 cycles, only slight scaling of the cement mortar paste separated from the aggregate appeared on the surface of the specimen. After 150 freeze–thaw cycles, it can be observed that the surface cement mortar around the aggregate gradually wrinkled and peeled off. With the increasing cycles, the appearance of the specimen is changed obviously. In addition, holes and cracks were observed easily in some areas of the specimen. After nearly 200 cycles, the cement mortar paste on the surface was completely flaked off, and the coarse aggregate is also completely exposed (see Figure 7 N = 200 cycles). Around 225–250 freeze–thaw cycles, the surface of the prismatic specimen was hardly wrapped with mortar, and the corners were severely damaged.

#### 3.1.2. Typical Failure Pattern of Cube Specimens

**Figure 8:**
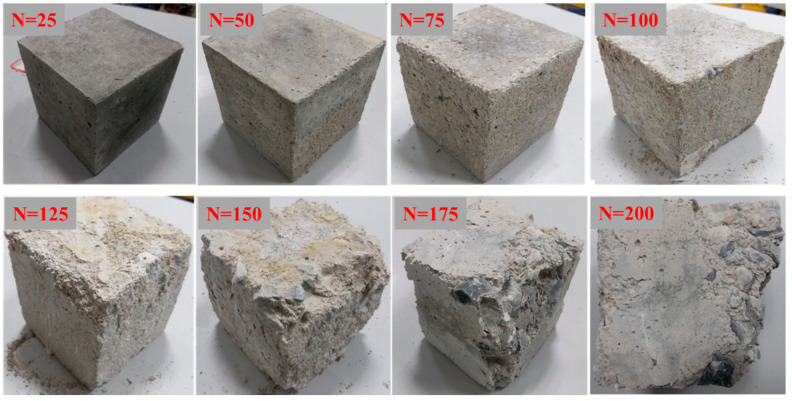
Typical failure characteristics of concrete cubic samples under different freeze–thaw cycles.

Figure 8 shows the appearances of cubic specimens after different freeze–thaw cycles. The surface of cubic specimens became rough, and the surface mortar was loosened gradually with the increasing of freezing–thawing cycles. Within 100 freeze–thaw cycles, only slight scaling of the cement mortar paste was observed on the surface of specimen. It can be clearly seen that the mortar was spalled at the corner of cubic specimens after 125 freeze–thaw cycles. Around 150 cycles, due to the periodic freezing and thawing process, the shape of cube specimen was no longer complete, and the mortar around the specimen had fallen off seriously. After 175–200 freeze–thaw cycles, with the further internal damage, the exposed coarse aggregate was peeled off.

The figure also shows that the cubic specimens after 200 freeze–thaw cycles present more serious surface scaling and more damage characteristics than the prism specimens after 200 freeze–thaw cycles. This can be explained in two ways: (1) influence of size effect; (2) since the ratio of surface area to volume around the cubic specimen is 1.33 times that of the prismatic specimen [42]. This also leads to the degree of frost damage gradually deepening.

### 3.2. Mass Loss Rate and RDME

#### 3.2.1. Mass Loss Rate

**Figure 9:**
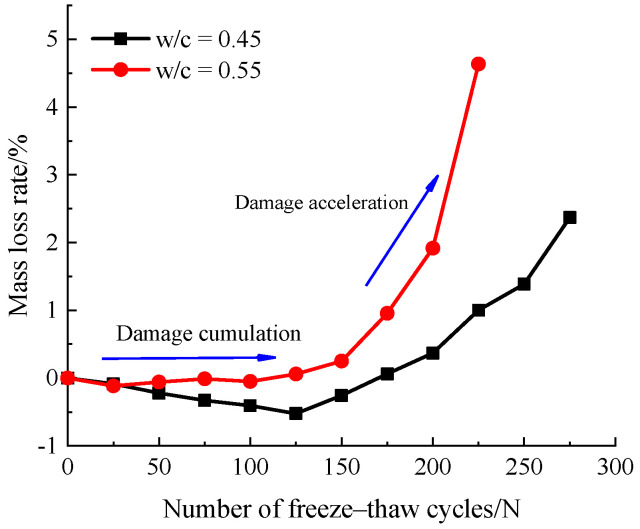
Variation of mass loss rate with different freeze–thaw cycles.

The relationship curves of mass loss rate of concrete specimens with the number of freeze–thaw cycles for different water–cement ratios are shown in Figure 9. As shown in this figure, the mass loss rate of both concrete specimens first decreased and then increased over the whole process of freeze–thaw cycles, which is accordance with results from Fang et al. [43]. For the w/c = 0.45 group, the mass of specimens increased slightly before 125 freeze–thaw cycles. This is attributed to the capillary pore water absorption and further hydration of cement paste. Subsequently, with the increase of freezing and thawing cycles, internal micro-cracks and pores of the concrete were gradually expanded and became connected which made the surface of specimen gradually crumbled. For the 0.55 water–cement ratio, the mass loss rate of the concrete prismatic specimens did not change significantly before 100 freeze–thaw cycles. However, after 150 freeze–thaw cycles, the mass loss rate of specimens has an obvious turning point, and it decreased more acutely than the degradation of the 0.45 water–cement ratio. At the 225 freeze–thaw cycles, the mass loss rate of concrete specimen with 0.45, 0.55 water–cement ratios were 1.004%, 4.636%, respectively. From Figure 9, it can be demonstrated that, specimens of 0.55 water–cement ratio failed after 250 cycles, while specimens of 0.45 water–cement ratio were still less than 5% after 300 cycles.

In addition, it is clear that the relationship between mass loss rate and freeze–thaw cycles exhibit the two distinct stages. In the first 0–125 freeze–thaw cycles, it can be identified that in the damage accumulation stage, the weight of concrete specimens shows a slightly increasing trend or is basically unchanged. However, after 150 freeze–thaw cycles, the mass loss rate increases significantly for the 0.45, 0.55 water–cement ratio specimens, which can be identified as the damage acceleration stage.

#### 3.2.2. Relative Dynamic Modulus of Elasticity

**Figure 10:**
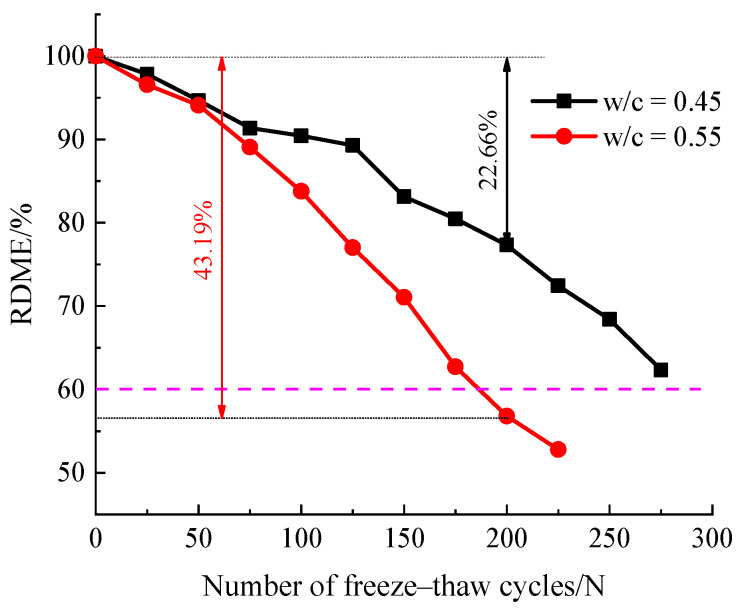
Variation of RDME with different freeze–thaw cycles.

The RDME can reflect the state of micro-crack development inside the concrete, the lower the RDME means the more serious degree of freeze–thaw damage. Figure 10 shows the degradation of the relative dynamic modulus of elasticity of the concrete specimens with the different water–cement ratio under the condition of freeze–thaw cycles. As shown in Figure 10, it can be seen that with the number of freeze–thaw cycles increased, the RDME of the concrete specimens decreased gradually. Before 75 freeze–thaw cycles, there was no significant difference in RDME between the two groups of concrete specimens. Nevertheless, after 100 freeze–thaw cycles, the RDME of the specimens with a water–cement ratio of 0.55 degraded more sharply than that of the specimens with a water–cement ratio of 0.45. At the end of 150 freeze–thaw cycles, the RDME of the specimens with water–cement ratios of 0.45, 0.55 are 83.12%, 71.05%, respectively. Around 200 freeze–thaw cycles, the RDME of concrete specimens decreased by 22.66%, 43.19%, respectively. The results indicate that the specimens of 0.55 water–cement ratio had reached the final number of freeze–thaw cycles, According to the standard GB/T 50082-2009. However, after 275 freeze–thaw cycles, the RDME of specimens with 0.45 water–cement ratio decreased by 37.6%. The results also indicated that there was significant difference in the rate of deterioration of different w/c ratios under the same number of freeze–thaw cycles; the higher the w/c ratio of concrete specimens, the faster the degradation rate.

The change of RDME can reflect the change of internal microstructure and overall compactness of concrete after frost damage, therefore, it can be used to characterize the degree of damage deterioration of freeze–thaw concrete [28,42]. It is worth noting that the mass loss of all mixtures was not apparent at the beginning of the freeze–thaw test. However, the RDME were always decreasing, which implies that the internal damage gradually developed and extended in the concrete specimens.

### 3.3. Compressive Strength

**Figure 11:**
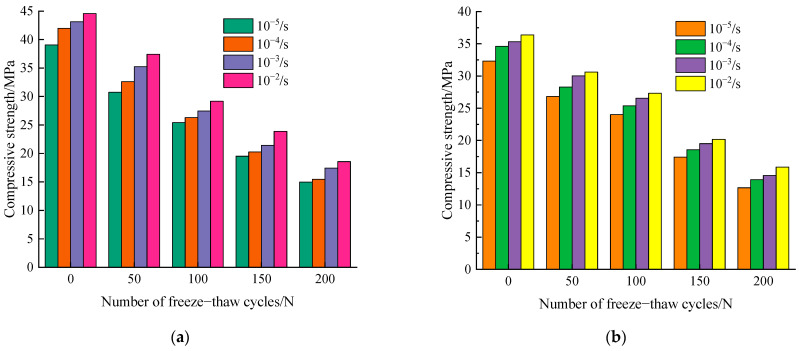
Dynamic compressive strength with different freeze–thaw cycles. (**a**) w/c = 0.45 and (**b**) w/c = 0.55.

In this study, the cubic compressive strength (*f_cu_*) was tested for characterizing the mechanical properties of concrete under different freeze–thaw cycles. Figure 11 shows the average dynamic compressive strength with different strain rates after frost damage. It is observed that the uniaxial compressive strength of concrete decreases with the increase of the number of freezing–thawing cycles, and under the same number of freezing–thawing cycles, uniaxial compressive strength increased gradually as strain rate increased from 10^−5^/s to 10^−2^/s. Furthermore, it is noted that the peak compressive strength of concrete shows a decreasing trend with the increase of freeze–thaw cycles at the same strain loading rate.

The results can be explained as follows: under the proposed static loading conditions, i.e., 10^−5^/s strain rate, the crack has sufficient time to select the weakest intersection to evolve and develop during concrete damage, and the crack sprouting occurs mostly at the weak intersection between mortar and aggregate [18]. Under the dynamic loading conditions, the specimen quickly reaches the peak compressive strength and the deformation rate is also faster. The material damage crack does not have sufficient time to select the weakest intersection to cause damage, but directly through the aggregate damage. It is noted that the greater the loading rate, the more aggregate is pulled off the rupture surface, thus enhancing the resistance of the material to external loading, resulting in the uniaxial ultimate compressive strength under dynamic action rather than under static action is increased [20].

#### 3.3.1. Specimens Failure Pattern

**Figure 12:**
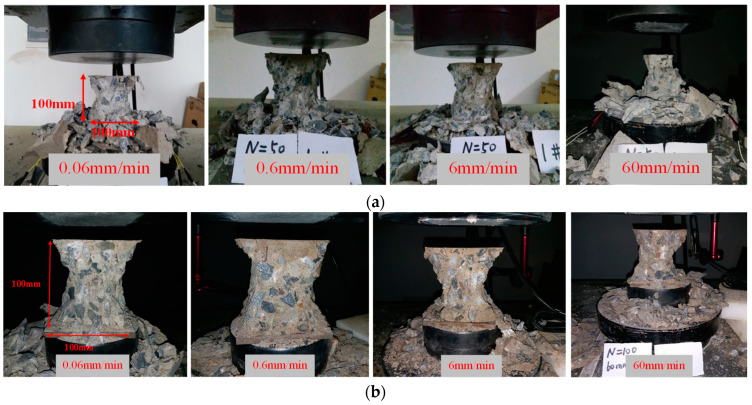

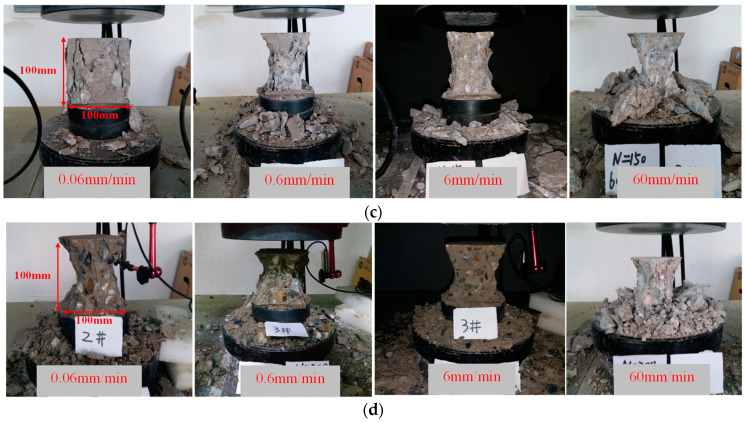
Failure modes with different strain rate and freeze–thaw cycles. (**a**) 50 freeze–thaw cycles, (**b**) 100 freeze–thaw cycles, (**c**) 150 freeze–thaw cycles, (**d**) 200 freeze–thaw cycles.

The failure patterns of cubic concrete specimens under different loading rates after different numbers of freeze–thaw cycles are given in Figure 12. According to this figure, the damage characteristics of concrete cubic specimens at different loading rates have significant variability. When the loading rate is slow, the cracks on the surface of the concrete specimen appear more and expand more evenly, roughly from the end of the oblique cracks gradually extended downward, a large number of bridging cracks, but the ruptured specimen can still rely on the friction between the rupture surface to transfer the load, so that the cubic specimen still has a certain load-bearing capacity. With the increase of the loading rate, the crack on the surface of the specimen is not fully expanded before it increases sharply, and the crack directly passes through part of the coarse aggregate, and the damage mode of the cubic specimen is an inverted cone shape. At this time, the splitting sound when the concrete specimen fractures and damages is more crisp and the broken state of the aggregate after rupture is more complete.

#### 3.3.2. Dynamic Increase Factor, DIF

Based on the obtained test data, the DIF is calculated for different working conditions.

(5) $$\alpha_{DIF}=\frac{f_{dc}}{f_{sc}}=a+b\mathrm{lg}\left({\dot{\varepsilon }}_{dc}/{\dot{\varepsilon }}_{sc}\right)$$

where *f_dc_* is the peak uniaxial compressive strength at the current strain loading rate, *f_sc_* is the uniaxial compressive strength at the quasi-static strain loading rate, and in this test, the quasi-static strain loading rate is taken as 10^−5^/s. a and b are the fitted material parameters, ${\dot{\varepsilon }}_{dc}$ is the dynamic strain rate corresponding to *f_dc_*, which is taken as 10^−4^/s, 10^−3^/s, 10^−2^/s, respectively.

**Figure 13:**
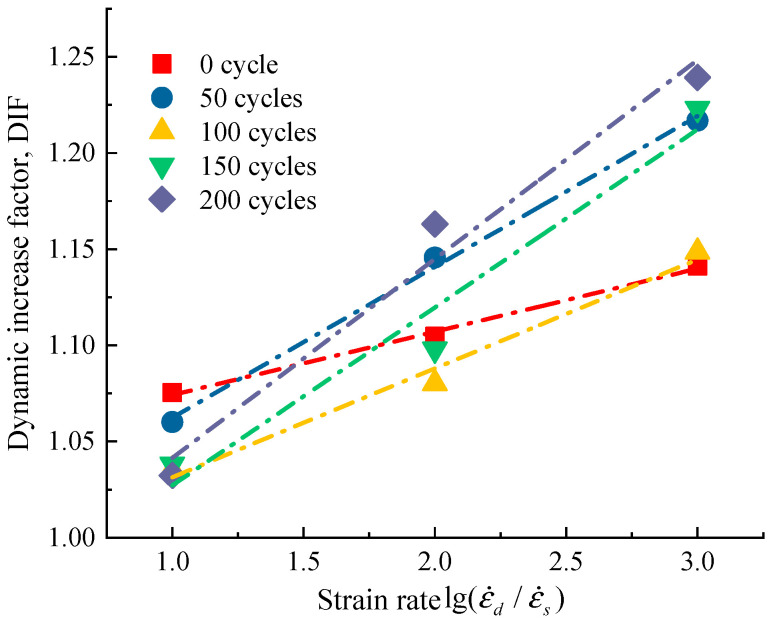
Variation of dynamic increase factor (DIF) and strain rate.

Taking the w/c = 0.45 as an example, Figure 13 presents the relationship between the DIF of uniaxial compressive strength with strain rate after frost damage. It can be visualized from the figure that the dynamic increase factor of concrete after freeze–thaw increases gradually with the increase of strain rate. The relationship between the DIF and the log strain rate was fitted according to Equation (4), we found a good linear relationship between the DIF and the log strain rate under the different freeze–thaw cycles. The regression equations and correlation coefficients of DIF and strain rate for different numbers of freeze–thaws are presented in Table 2. These regression equations are similar to that proposed by Han et al. [20]. As can be seen in Table 2, the slope of the fitted regression equation gradually increases after frost damage, which indicates that the increase of freeze–thaw cycles improves the rate sensitivity of concrete.

### 3.4. Flexural Strength and Splitting Tensile Strength

**Figure 14:**
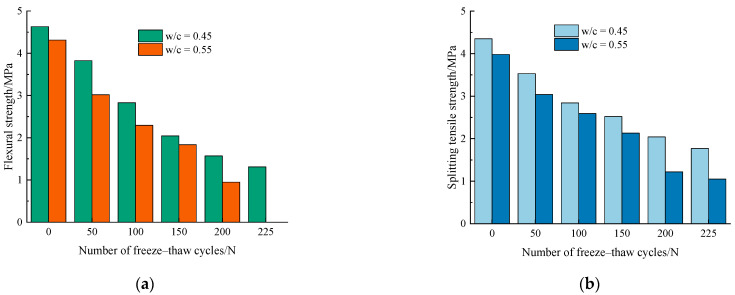
Variation of mechanical properties of concrete specimens under frost damage. (**a**) Flexural strength, (**b**) splitting tensile strength.

In this test, the flexural strength (***f_f_***) and splitting tensile strength (***f_st_***) tests were also performed to evaluate the durability of the concrete specimens after freeze–thaw cycles. The average experiment results of ***f_f_*** and ***f_st_*** are listed in Figure 14a,b, respectively. It was observed that both ***f_f_*** and ***f_st_*** decreased continuously with the increase of the number of freeze–thaw cycles. This phenomenon might probably be due to the internal damage gradually accumulated during the freeze–thaw cycles, which indicates the micro-cracks have gradually expanded and penetrated in the concrete specimen.

As shown in Figure 14a, the flexural strength of concrete specimens for both water–cement ratios decreased gradually with the increase in the number of freeze–thaw cycles. For concrete specimens with 0.55 water–cement ratio, the flexural strength decreased significantly after 50 times of freezing–thawing. After 100 cycles of freezing and thawing, it is noted that the flexural strengths of the specimens with w/c = 0.45 and 0.55 are 2.83 MPa, 2.29 MPa, respectively. With the accumulation of the degree of freeze–thaw damage, after 200 freeze–thaw cycles, the concrete specimen with 0.55 water–cement ratio has a flexural strength of only 0.95 MPa, which has basically lost its load-bearing capacity.

Figure 14b shows the relationship between splitting tensile strength and freeze–thaw cycles for different water–cement ratios. Similar to the deterioration law of flexural strength with the number of freeze–thaws, the splitting tensile strength of different water–cement ratios also decreases after frost damage.

In summary, the flexural strength and splitting tensile strength showed an obvious two-stage change pattern subject to freeze and thaw damage. Before 100 freeze–thaw cycles, both of them decreased rapidly. After that, the loss of strength obviously slowed.

**Figure 15:**
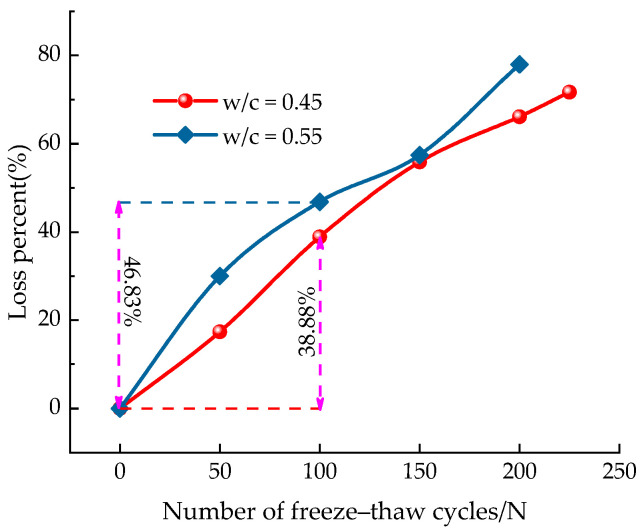
Variation of the loss rate of ***f_f_*** of concrete specimens with different water–cement ratios under freeze–thaw cycles.

Figure 15 depicts the flexural strength loss rate of concrete specimens with different water–cement ratios after freeze–thaw cycles. According to this figure, it can be observed that the flexural strength loss rate of the two groups of concrete specimens have the similar trend. With the increase of the number of freeze–thaw cycles, the loss rate of flexural strength tended to increase significantly. Moreover, there was an approximate linear relationship between the rate of loss of flexural strength and the number of freeze–thaw cycles. After 100 freeze–thaw cycles, the flexural strength of 0.45 water–cement ratio concrete specimens and 0.55 water–cement ratio concrete specimens decreased by 38.88% and 46.83%, respectively. After being subjected to 200 freeze–thaw cycles, the flexural strength loss rate of 0.55 water–cement ratio concrete is nearly 80%. Moreover, it is worth noting that the loss rate of flexural strength is more obvious compared to the RDME, which is mainly due to the freeze–thaw action that makes the concrete specimens gradually crisp inside and the micro-cracks gradually expand and penetrate, leading to the reduction of the overall compactness.

## 4. Microstructure Characterization Based on NMR Porosity

### 4.1. NMR T_2_ Distribution

**Figure 16:**
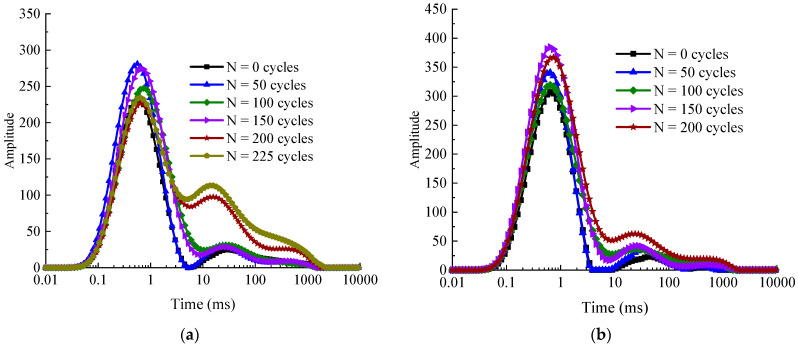
Changes of T_2_ distribution curves of concrete samples after different numbers of freeze–thaw cycles. (**a**) w/c = 0.45 and (**b**) w/c = 0.55.

Figure 16 illustrates the typical *T*_2_ transverse relaxation time distribution curves of the specimens with different water–cement ratios under different freeze–thaw cycles. From the figure, it can be seen that the transverse relaxation time *T*_2_ distribution curves contains three peaks, which corresponds to the variations of micro-pore, meso-pore, and macro-pore from left to right, respectively. The area occupied by the leftmost peak is the largest, which indicates that the micro-pores occupy the large proportion of the total number of pores. At the beginning and early stages of freeze–thaw cycles (0–50 cycles), the 3rd peak is not obvious, and with the increase of the number of freeze–thaw cycles, the pore structure of the concrete core sample changes, and the proportion of the 2nd and 3rd peaks gradually increases, while the 1st peak gradually decreases, which means that the freeze–thaw cycles make the internal micro-pores gradually expand and penetrate, making the micro-pores gradually evolve into medium and large pores. After 200 freeze–thaw cycles, the increase of the 2nd and 3rd peaks on the right side of the *T*_2_ relaxation time curve was significantly higher than that of the 1st peak, indicating that the micro-pores and meso-pores inside the concrete continuously expand and penetrate into macro-pores. This indicates that with the accumulation of frost damage, the external moisture continues to migrate into the newly generated pores, generating the internal frost heaving pressure and osmotic pressure of concrete increasing, the pore structure continues to deteriorate, small pores continue to develop, expand, and even penetrate, forming medium and large pores [36,44].

### 4.2. Pore Structure

Concrete is a typical porous material, and the internal porosity and pore size distribution are closely related to the mechanical properties. According to the results of [45], the pore inside the concrete can be classified into the following four grades, specifically, harmless pores (pore diameter less than 20 nm), less harmful pores (pore diameter between 20 and 50 nm), harmful pores (pore diameter greater than 50 nm and less than 200 nm), and multi-harmful pores (pore diameter greater than 200 nm), respectively. Generally, harmless pores have no effect on the macroscopic mechanical properties and durability of concrete; less harmful pores have a minor effect on the mechanical properties; harmful pores have a negative effect on the performance of concrete; when the pore size exceeds 200 nm, it has a serious effect on the mechanical properties of concrete.

**Figure 17:**
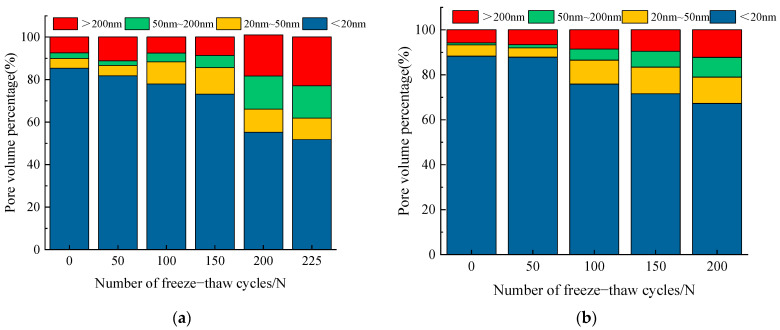
Pore size distribution of concrete with different w/c ratios after freezing–thawing damage. (**a**) w/c = 0.45; (**b**) w/c = 0.55.

In Figure 17, the pore volume percentages of concrete core samples with different water–cement ratio after frost damage are demonstrated. It can be observed that the percentage of harmless pores is much higher than the percentage of the other three classes of pores before the start of freeze–thawing. With the increase of the number of freeze–thaw cycles, both the porosity and pore size distribution of specimens were changed obviously. After 100 freeze–thaw cycles, the proportion of harmful pores and more harmful pores gradually increases, while the proportion of harmless pores gradually decreases. Subsequently, with the development and expansion of the micro-cracks caused by freeze–thaw cycles, the internal structure of concrete specimens was no longer compact. As a result, the periodic freeze–thaw cycles cause a large number of harmful pores and multi-harmful pores in the concrete [45,46], which greatly destroys the durability of concrete structure. Therefore, the RDME and its mechanical properties of concrete gradually declined.

### 4.3. Quantification of Concrete Porosity with LF-NMR

**Figure 18:**
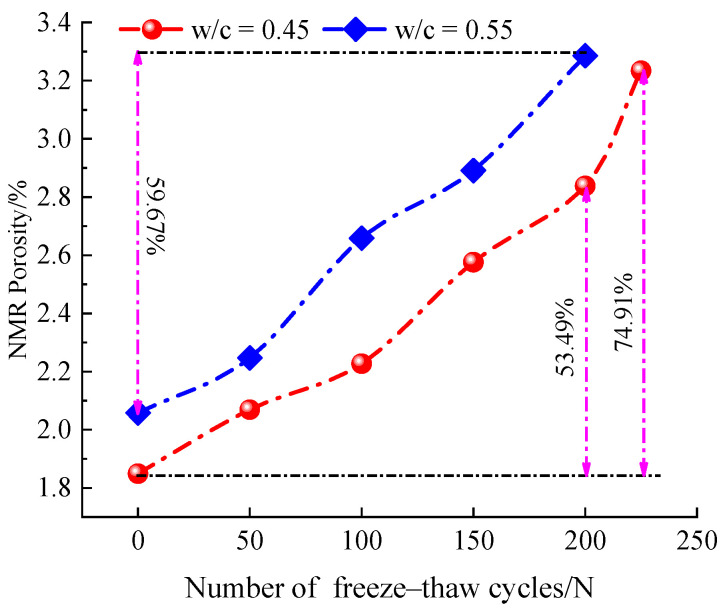
NMR porosity of concrete samples after different freeze–thaw cycles.

The porosity is a significant property used to quantify the pore structure of concrete. Figure 18 demonstrates the porosity variation of concrete cylinder specimens with different w/c ratios after different freeze–thaw cycles. It can be seen that the porosity of concrete core samples gradually increased with the increase of the number of freeze–thaw cycles, which implies the internal damage of concrete samples becomes more and more serious. After 200 freeze–thaw cycles, the NMR porosity of 0.45 and 0.55 water–cement ratios increased by 53.49% and 59.67%, respectively. However, the porosity of 0.45 water–cement ratios showed significantly faster growth after 225 freeze–thaw cycles.

It can also be seen from the figure that at the early stage of freeze–thaw (0–25 cycles), the porosity hardly changed, while at the stage of 50–125 freeze–thaw cycles, the growth of NMR porosity was significantly accelerated. Subsequently, at the stage of 125–175 freeze–thaw cycles, the growth of porosity was slower. The analysis concluded that at the beginning of freeze–thaw cycles, the concrete is relatively compact inside and the overall degree of compactness is high; as the number of freeze–thaw cycles increases, the repeated action of periodic freezing and expansion forces and hydrostatic pressure on the pores inside the concrete causes the pores inside the concrete to gradually expand, penetrate, and form connected pores [45]. At the 125–175 freeze–thaw stages, the slower growth of porosity may be due to the expansion and penetration of meso-pores and macro-pores, and sufficient space inside the concrete can all offset the freeze swelling force generated by the freeze–thaw process.

**Figure 19:**
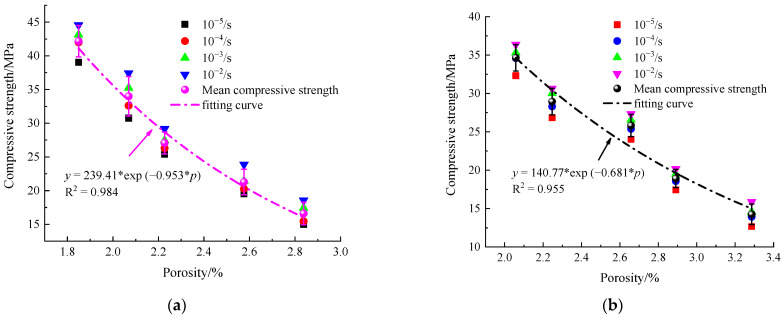
Relationship between compressive strength of concrete with different w/c ratio and porosity. (**a**) w/c = 0.45; (**b**) w/c = 0.55.

In previous studies, most of the literature has focused on the variation of macroscopic mechanical properties of concrete under the condition of freeze–thaws cycles. In fact, the microscopic pore structure and pore size distribution inside concrete are closely related to the macroscopic mechanical properties and durability [23,24,44]. Therefore, it is necessary to establish a damage variable based on pore structure to describe the evolution of mechanical properties of concrete under different freeze–thaw cycling conditions. Based on the test results, the relationship curve between NMR porosity and compressive strength is established, as shown in Figure 19. From the figure, it can be seen that the compressive strength decreased with the increase of porosity as exponential function, which coincides with the results from Kumar et al. [47].

### 4.4. Establishing Freeze–Thaw Damage Variables with Porosity Changes

The most direct result of the damage caused by freeze–thaw cycles on concrete is the increase of porosity. Therefore, the change in porosity is introduced as the basis for establishing the damage variable *D* to evaluate the damage of concrete with different water–cement ratios after frost damage, and this damage variable can quantitatively reflect the degree of damage to the internal microstructure of concrete specimens, and the relationship between the damage variable *D* and porosity can be defined as

(6) $$D=\frac{p_{n}-p_{0}}{1-p_{0}}$$

where $p_{n}$ is the NMR porosity after *n* freeze–thaw cycles, %; $p_{0}$ is the initial NMR porosity before the freeze–thaw cycles, %.

**Figure 20:**
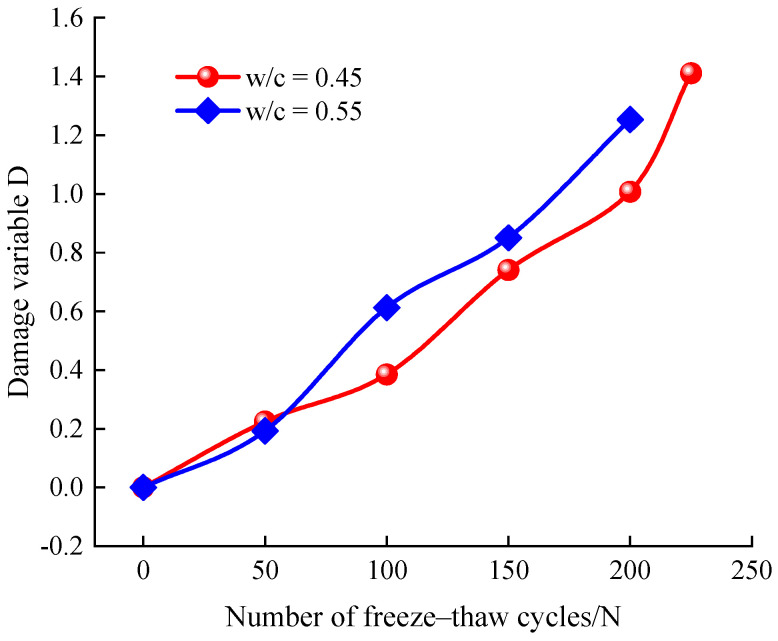
Relationship between damage variable *D* of concrete specimens and freeze–thaw cycles.

The damage variables of porosity of concrete after different number of freeze–thaw cycles were calculated according to Equation (5), as shown in Figure 20. As can be seen from the figure, with the increase of the number of freeze–thaw cycles, the damage variables of concrete specimens with both water–cement ratios gradually increased, indicating that the freeze–thaw action made the concrete damage gradually intensify, but there is a certain difference in the increased rate of the damage degree of specimens with different water–cement ratios, and the damage degree of specimens with 0.55 water–cement ratio was greater than the damage deterioration degree of 0.45 water–cement ratio under the same number of freeze–thaw cycles.

**Figure 21:**
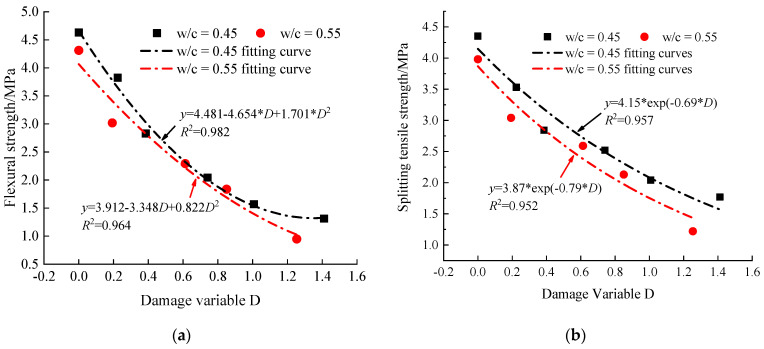
Relationship between mechanical properties of concrete specimens with different w/c ratio and damage variable. (**a**) Flexural strength and damage variable; (**b**) splitting tensile strength and damage variable.

In order to further analyze and explore the quantitative relationship between microscopic pore structure changes and macroscopic damage characteristics of concrete, a fitted regression analysis was performed on the porosity damage variables and macroscopic damage characteristics indicators. As shown in the Figure 21a,b, it can be observed that the flexural strength (***f_f_***) and splitting tensile strength (***f_st_***) of concrete specimens gradually decreased with the increase of the porosity damage. This indicates that the degree of deterioration gradually increases with the increase of the number of freeze–thaw cycles, and the accumulation of damage to the microscopic pore structure leads to different degrees of deterioration of the macroscopic mechanical and mechanical characteristics of the concrete specimens.

According to Figure 21a, it can be seen that the regression equations of flexural strength ***f_f_*** and damage variable D all satisfy the quadratic parabolic equation, and the correlation coefficient R^2^ is greater than 0.964. This fully indicates that there is a good quantitative correspondence between the microstructural changes of concrete pores and macroscopic damage characteristics under different freeze–thaw cycles.

## 5. Further Discussion

**Figure 22:**
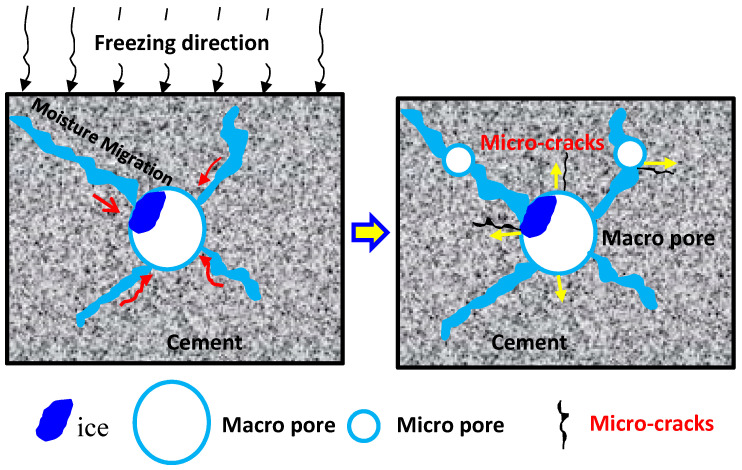
Schematic diagram of moisture migration in different sized pores during freezing.

The main reason for the concrete failure under freezing and thawing cycles is the generation, expanding, and developing of micro-cracks inside the concrete. The test results of Luan et al. [48] showed that the water absorption of concrete increased significantly after freezing and thawing, and the degree of water saturation increased rapidly. When the temperature drops below 0 °C, the moisture in capillary pores inside the concrete phases into ice and expands by about 9% of its volume [3,10]. During the freezing process, unfrozen water gradually migrates around the frozen macro-pores. The migration of the pore solution eventually creates hydrostatic and osmotic pressure. When the expansion force exceeds the tensile strength of the capillary wall, the micro-cracks start to expand around and gradually penetrate in cement. Under the action of hydrostatic pressure, the weak surface of large pores will be destroyed first and then generate secondary pores around the large pores. Based on the test results, a conceptual model for the moisture migration in different sized pores during the freezing process induced concrete deterioration is shown in Figure 22.

According to the experimental results, all of the RDME, dynamic compressive strength, flexural strength, and splitting tensile strength decrease with the increase of freeze–thaw cycles, which is shown in Figure 10, Figure 11 and Figure 14, respectively. This is because the pores penetrate each other and form a continuous seepage channel, and finally lead to macroscopic damage (as shown in Figure 16 and Figure 17), which is in accordance with results from [31,34,35,46]. Moreover, the water–cement ratio has an important effect on the degradation of concrete strength under freeze–thaw cycles. The concrete specimens with 0.45 water–cement ratio show a better frost resistance (see Figure 9, Figure 10, Figure 15, Figure 18 and Figure 20). The results can explain that the lower the water–cement ratio, the better the internal compactness of the concrete. Then, the corresponding resistance to permeability improves and the saturation of the concrete surface reduces. As a result, the lower water–cement ratio shows higher frost resistance, which agrees well with other literature [39,44,45].

## 6. Conclusions

In this study, the dynamic evolution of mechanical properties, durability, and pore structure of two types of water–cement ratio (w/c = 0.45, w/c = 0.55) concrete under freeze–thaw cycles were systematically investigated, and the degradation mechanism of concrete was explored based on porosity damage variables. Based on the experimental results analysis above, the following conclusions were drawn:

(1) With the increase of the number of freeze–thaw cycles, the appearance characteristics of concrete became more and more rough, and the apparent damage of cubic specimens was more serious than that of prism specimens. The change rule of concrete mass loss varying with freeze–thaw cycles shows obvious two-stage distribution. Before 125 freeze–thaw cycles, the mass increases slightly due to the capillary pore water absorption and further hydration of cement paste. After that, the mass loss rate increases gradually become larger.
(2) The concrete specimen with 0.45 water–cement ratio has better performance of frost resistance and durability. The RDME, flexural strength, and splitting tensile strength of concrete decrease 22.66%, 66.1%, and 53.1% after 200 freeze–thaw cycles, respectively.
(3) The results of dynamic loading tests show that there is a good linear relationship between the DIF and the log strain rate under the different freeze–thaw cycles. With the increase of strain rate, the peak stress of concrete with the same degree of freeze–thaw deterioration gradually increases. In addition, the sensitivity of the peak stress to the strain rate gradually increases with the increase of the number of freeze–thaw cycles.
(4) The porosity as well as the proportion of meso-pores and macro-pores gradually increase, which indicates that the deterioration of concrete interior is gradually serious with the increase of the number of freeze–thaw cycles. After 200 freeze–thaw cycles, the NMR porosity of 0.45 and 0.55 water–cement ratios increased by 53.49% and 59.67%, respectively.
(5) Finally, a damage model based on the porosity variation was established to quantitatively describe the degradation law of macroscopic mechanical properties, which matches well with the experimental results.
(6) The results in this work can provide data support and reference for the durability prediction and safety assessment of concrete structures in cold regions, such as concrete dams, sluices, and canals. To improve the durability and service life of the concrete structures in cold climates, the porosity and water–cement ratio should be reduced.

## Figures and Tables

**Table 1:** Mix proportion of concretes investigated in this study (kg/m^3^).

Water–Cement Ratio	Cement	Coarse Aggregate	Sand	Water	Superplasticiser
0.45	324.78	1253.94	675.12	146.15	2.598
0.55	265.72	1292.28	695.85	146.15	2.126

**Table 2:** Regress equations of the relationship between strain rate and DIF under different freeze–thaw cycles.

Freeze–Thaw Cycles/N	Regress Equations	R^2^
N = 0	$\alpha_{DIF}=1.041+0.033\mathrm{lg}\left({\dot{\varepsilon }}_{dc}/{\dot{\varepsilon }}_{sc}\right)$	0.996
N = 50	$\alpha_{DIF}=0.984+0.078\mathrm{lg}\left({\dot{\varepsilon }}_{dc}/{\dot{\varepsilon }}_{sc}\right)$	0.997
N = 100	$\alpha_{DIF}=0.975+0.056\mathrm{lg}\left({\dot{\varepsilon }}_{dc}/{\dot{\varepsilon }}_{sc}\right)$	0.986
N = 150	$\alpha_{DIF}=0.934+0.092\mathrm{lg}\left({\dot{\varepsilon }}_{dc}/{\dot{\varepsilon }}_{sc}\right)$	0.960
N = 200	$\alpha_{DIF}=0.938+0.103\mathrm{lg}\left({\dot{\varepsilon }}_{dc}/{\dot{\varepsilon }}_{sc}\right)$	0.977

## Data Availability

Data is contained within the article.
